# Evaluation of Avulsion-Induced Neuropathology in Rat Spinal Cords with ^18^F-FDG Micro-PET/CT

**DOI:** 10.1371/journal.pone.0127685

**Published:** 2015-05-26

**Authors:** Ze-Min Ling, Ying Tang, Ying-Qin Li, Hao-Xuan Luo, Lin-Lin Liu, Qing-Qiang Tu, Li-Hua Zhou

**Affiliations:** 1 Department of Anatomy, Zhongshan School of Medicine, Sun Yat-sen University, No. 74 Zhongshan Road 2, Guangzhou, 510080, P.R. China; 2 Small Animal Molecular Imaging Center, Laboratories of Translational Medicine and Clinical Research, Sun Yat-sen University, No. 74 Zhongshan Road 2, Guangzhou, 510080, P.R. China; Inserm, FRANCE

## Abstract

Brachial plexus root avulsion (BPRA) leads to dramatic motoneuron death and glial reactions in the corresponding spinal segments at the late stage of injury. To protect spinal motoneurons, assessment of the affected spinal segments should be done at an earlier stage of the injury. In this study, we employed ^18^F-FDG small-animal PET/CT to assess the severity of BPRA-induced cervical spinal cord injuries. Adult Sprague-Dawley rats were randomly treated and divided into three groups: Av+NS (brachial plexus root avulsion (Av) treated with normal saline), Av+GM1 (treated with monosialoganglioside), and control. At time points of 3 day (d), 1 week (w), 2 w, 4 w and 8 w post-injury, ^18^F-FDG micro-PET/CT scans and neuropathology assessments of the injured spinal roots, as well as the spinal cord, were performed. The outcomes of the different treatments were compared. The results showed that BPRA induced local bleeding and typical Wallerian degeneration of the avulsed roots accompanied by ^18^F-FDG accumulations at the ipsilateral cervical intervertebral foramen. BPRA-induced astrocyte reactions and overexpression of neuronal nitric oxide synthase in the motoneurons correlated with higher ^18^F-FDG uptake in the ipsilateral cervical spinal cord during the first 2 w post-injury. The GM1 treatment reduced BPRA-induced astrocyte reactions and inhibited the de novo nNOS expressions in spinal motoneurons. The GM1 treatment also protected spinal motoneurons from avulsion within the first 4 w post-injury. The data from this study suggest that ^18^F-FDG PET/CT could be used to assess the severity of BPRA-induced primary and secondary injuries in the spinal cord. Furthermore, GM1 is an effective drug for reducing primary and secondary spinal cord injuries following BPRA.

## Introduction

Brachial plexus root avulsion (BPRA), which leads to the paralysis of the ipsilateral upper limbs, is the most serious form of peripheral nerve injury [1]. When treating BPRA, it is very hard to re-establish reinnervation of the intrinsic muscles in the hand. Even with successful surgical reconnection of the injured brachial plexus, the death of motoneurons often prevents successful reinnervation [2]. Therefore, rehabilitation of the motor functions in the upper limbs depends on the regenerative capacity of the affected motoneurons [3, 4]. In previous laboratory studies, avulsion-induced motoneuron injury was triggered by the deprivation of the target-derived neurotrophic factors [5–7], followed by secondary glial reactions [8, 9] and oxidative stress [10–13] in the affected spinal segments. All these cellular reactions corresponded with motoneuron death. However, regeneration of the avulsed axons of the affected motoneurons also occurred. All these pathological changes and the regenerative processes have proved to cause metabolism changes in spinal cord tissues [14]. Therefore, we believe that surgical reconnection should be assessed in vivo based on recovery of the proximal and distal parts of the avulsed nerve roots as well as on pathological changes in the corresponding spinal segments.

In vivo positron emission tomography (PET)/Computed Tomography (CT) images could be helpful in deciding the appropriate surgical intervention for BPRA injury.

Combining non-invasive positron emission tomography (PET) with [^18^F]fluoro-2-deoxy-D-glucose (^18^F-FDG), a widely used PET tracer, has allowed in vivo imaging of specific biological pathways, such as increased glucose utilization in tumour cells [15], the high uptake of inflammatory cellular elements and blood supply in the clinic. In laboratory studies, ^18^F-FDG micro-PET-CT (computed tomography) imaging, due to its inherent imaging characteristics, has allowed scientists to investigate whole body metabolic activity and acquire images reflecting quantitative metabolic information in regions of interests (ROIs) in small-animal models of different diseases [14, 16, 17]. Our recent study demonstrated that changes in ^18^F-FDG micro-PET-CT images can reflect the size of ischemia and neuronal loss in the prefrontal cortex 2 weeks after cerebral ischemia/reperfusion of adult rats [18]. In the present study, we tested for changes in avulsion-induced ROIs in the spinal cord with ^18^F-FDG micro-PET-CT imaging following BPRA of adult rats in vivo, and we determined the pathological changes in the corresponding spinal segments after sacrificing the animals.

To ensure the feasibility of ^18^F-FDG PET/CT imaging as a non-invasive diagnostic tool for BPRA, we used monosialoganglioside (GM1) to treat BPRA-induced spinal cord injuries. Gangliosides are compounds that occur naturally in cell membranes. Laboratory studies have suggested that gangliosides have beneficial effects on nerve re-growth and act as growth factors for central cholinergic neurons [19, 20]. Additionally, several clinical trials in humans have revealed that monosialoganglioside (GM1) can improve locomotor function of patients suffering from spinal cord injury [21, 22]. Therefore, we employed GM1 treatment in the hope that it could improve neuronal microenvironments and lead to additional neuronal survival following BPRA-induce injury.

## Materials and Methods

### Animals and Ethics Statement

All animal procedures were conducted with the approval of the Animal Research Ethics Board at Zhongshan School of Medicine at Sun Yat-sen University (protocol number: SCXK 2011–0029), and the animal procedures were performed in accordance with the Chinese National Institutes of Health Guide for Animal Care. Ninety male Sprague-Dawley rats (200–250 g) were used in this study. They were randomly divided into three groups: BPRA treated with GM1 (Av+GM1, n = 30), BPRA treated with normal saline (Av+NS group, n = 30), and control (Sham, n = 30). The rats were housed with five animals per cage under constant environmental conditions and a 12-hour light-dark cycle. Food and water were provided ad libitum.

### Surgery and GM1 Treatment

According to previous publications [23, 24], the microsurgery was performed to generate an animal model of right brachial plexus avulsion. Briefly, the animals were anesthetized intraperitoneally with 10% chloral hydrate (350 mg/kg). In the supine position, the right brachial plexus was exposed, and the 5 spinal roots, including C5–8 and T1, were isolated and pulled out one by one with a microhemostatic forceps. The distal ends of each avulsed nerve root were removed and examined under a microscope. Successful injury was confirmed by checking the avulsed ventral root and the avulsed dorsal root with its ganglion. For rats in the sham control group, the right brachial plexus was also explored and isolated but not avulsed. The muscle and skin were sutured in layers following avulsion. The animals were placed in a heated recovery chamber and returned to their cage once they were awake. All microsurgical procedures were conducted aseptically in accordance with Chinese National Health and Medical Research Council (NHMRC) animal ethics guidelines. Immediately following the surgery, the rats in the Av+GM1 group were treated with a daily intraperitoneal injection of 3 ml/kg GM1 (10 mg/ml, Shandong Qilu Pharmaceutical, China), whereas the rats in the Av+NS group were treated with 1.0 ml normal saline. During the 8 weeks of the study, none of the rats died.

### PET/CT Scanning of the Rat Spinal Cord

The ^18^F-FDG micro-PET/CT examination was performed on 45 of the rats in vivo followed by in vitro immunohistochemistry examination at time points of 3 d (n = 15, in each group), 1 w (n = 15, in each group), 2 w (n = 15, in each group), 4 w (n = 10, in each group), and 8 w (n = 5, in each group) following BPRA and GM1 treatment. The ^18^F-FDG micro-PET/CT scan of the rats and the image analyses were carried out according to procedures from previous studies [14,18]. The rats, after fasting for 12 hours and having no access to water 6 hours before the experiments, were anesthetized with an intraperitoneal injection of 10% chloral hydrate (350 mg/kg). The ^18^F-FDG [37 kBq (1 μCi)/g], provided by the First Affiliated Hospital of Sun Yat-sen University (Guangzhou, China), was injected via the tail vein. Forty minutes following the ^18^F-FDG injection, the rats were fixed in a prone position and scanned with a small-animal PET/CT scanner using Inveon software (Siemens Medical Solutions, Germany). After setting the scanning parameters (80 kV, 500 μA, target detection range from head to diaphragm according to the Topogram), static image acquisition was performed for 20 min. Low-dose spiral CT detection was conducted for the first 5 minutes, and then, PET scanning was performed for 15 minutes in 3D scanning mode with two bed positions. All PET images were corrected for emission scatter and attenuation by using CT, and the images were reconstructed with the two-dimensional ordered-subsets expectation maximum (OSEM). The animals were visually monitored for breathing and any other signs of distress throughout the imaging period.

**Fig 1:**
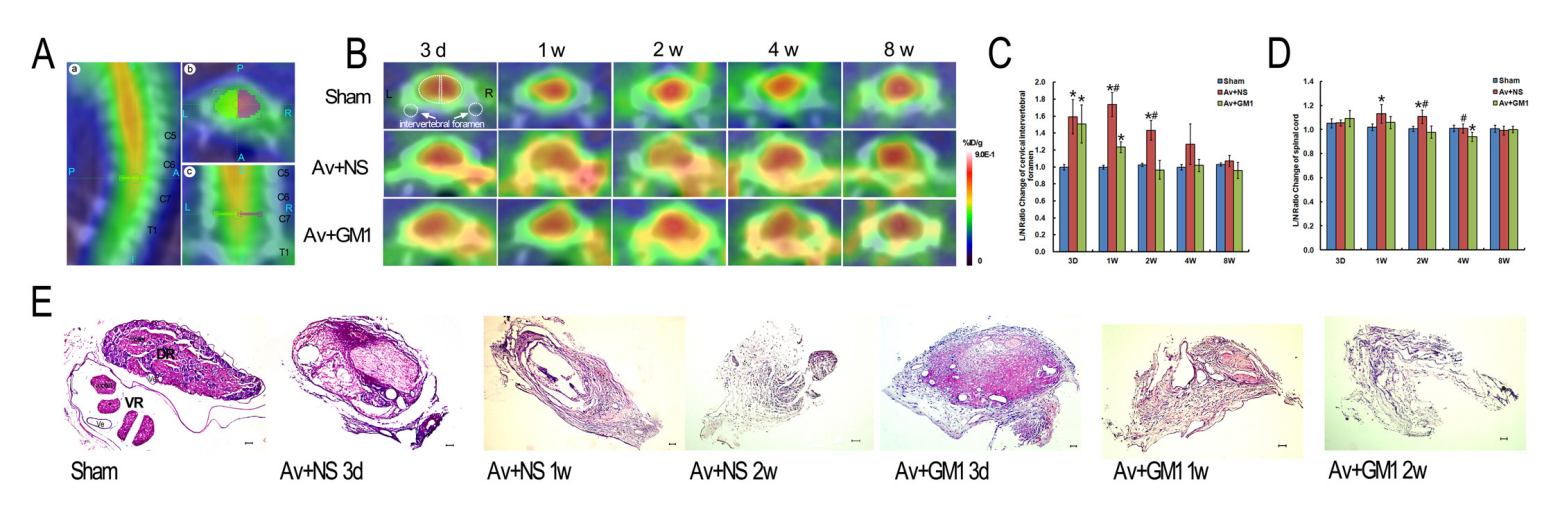
Higher ^18^F-FDG accumulation in ipsilateral cervical intervertebral foramen and spinal cord was reduced by GM1 treatment in the rats with a right brachial plexus root avulsion injury at 3 d, 1 w, 2 w, 4 w, and 8 w post-injury. (A): C7–C8 spinal segment located in sagittal (A,a), axial (A,b), and coronal (A,c) sections [P = Posterior, A = Anterior, R = Right, L = Left, S = Superior, I = Inferior]. (B): Time-dependent serial PET/CT scans of C7–C8 spinal segments of the rats in the Sham (sham control), Av+NS (BPRA with normal saline treatment), and Av+GM1 (BPRA with GM1 treatment) groups. Images of the C7–C8 spinal segments are shown in cross sections (A,b). The scale was set according to signal intensity. (C-D): Semiquantitative analysis of the I/C ratios of ^**18**^F-FDG uptake at the cervical intervertebral foramen (C) and spinal cord (D). *p < 0.05 compared to the Sham group, #*p* < 0.05 compared to the Av + GM1 group. I/C ratio = mean counts per pixel of lesion ROIs/mean counts per pixel of the contralateral normal area [indicated by the white dotted semi-oval or the white dotted circle in Fig 1B]. (E): The histology of the distal ends of the avulsed and un-avulsed C8 nerve roots with HE staining. In the sham control rats, the dorsal and ventral roots were completely separated by the nerve sheaths, and the axons were well organized. In rats from the Av+NS and Av+GM1 groups, the arrangement of the dorsal and the ventral roots disappeared, and bleeding, degenerated axons, and fibrosis masses occurred. There was comparatively less bleeding and earlier gliosis in the Av+GM1 group. Scale bar = 50 μm. [DR: dorsal roots; VR: ventral roots; Ve: vessel].

By using Inveon research workplace 4.1 software, the ^18^F-FDG absorption rate was expressed as the percent of the injected dose per gram of body weight (%ID/g). For each small-animal PET scan, the ROIs were delineated in 3 dimensions on the CT images. Co-registration of the PET images was used to ensure consistency in anatomic localization. In each image plane, three-dimensional ROIs were measured at a size of 2.261 mm*3.391 mm*2.261 mm over the ipsilateral C5–T1 cervical spinal segments and the contralateral normal spinal cord tissues. ROIs at a size of 1.582 mm*1.582 mm*2.261 mm were also measured over the ipsilateral C5–T1 intervertebral foramen and the contralateral normal intervertebral foramen. Then, the ROIs of the ipsilateral (right) and the contralateral (left) sides of the spinal cord were decay-corrected against the whole-body axial images (Fig 1). Meanwhile, ROIs of 2.261 mm*2.261 mm*2.261 mm for the thoracic spinal cord were collected from images of the upper thoracic spinal cord (T3–T4). The spinal disk and intervertebral space (for the T2 and CT images) were used to define the height of the spinal cord segment. The average radioactivity concentration within the ipsilateral cervical spinal cord was obtained from mean pixel values, normalized to the mean pixel values of the contralateral cervical spinal cord, and expressed as a percentage. ROIs for each region were represented as the average of 3 ROIs by repeating the measurements 3 times. For semiquantitative analysis, the lesion (ipsilateral)-to-normal (contralateral) homologous contralateral (I/C) ratio was calculated using the following formula: mean counts per pixel of lesioned region of interest/mean counts per pixel of the contralateral normal area.

### Histology Staining of the Injured Spinal Roots

At the end of 3 d, 7 d and 2 weeks post-injury, morphological examinations of the avulsed ventral and dorsal roots of the right brachial plexus were conducted on 45 rats (n = 5 in each group at each time point). The procedure for the routine haematoxylin and eosin (HE) staining was performed according to the protocol from a previous publication [4]. All the animals received a lethal dose of chloral hydrate and were transcardially perfused with normal saline followed by 4% paraformaldehyde (PFA) in 0.1 M phosphate buffer (PB, pH 7.4). The dorsal roots and ventral roots were removed. The roots were post-fixed in 4% PFA followed by immersion in 30% (v/v) sucrose in PBS at 4°C overnight. Transverse sections of the ventral and dorsal roots (10 μm) were cut on a cryostat, and HE staining was used to examine the nerve architecture and general histological details.

### Immunohistochemistry

For the morphological examinations, fifteen of the animals were sacrificed approximately 4 hours after their final FDG scans at the end of 2 w, 4 w, and 8 w (n = 5, in each group at each sacrifice time point). The rats were anesthetized with a lethal dose of chloral hydrate then perfused intracardially with normal saline at room temperature followed by a fixative containing 4% paraformaldehyde in 0.1 M phosphate buffer (pH 7.4) at 4°C. The spinal cords were harvested, post-fixed in 4% paraformaldehyde and subsequently cryoprotected in 30% phosphate-buffered sucrose overnight. Frozen transverse sections (35 μm) were cut with a microtome and collected in 0.01 M phosphate-buffered saline (PBS; pH 7.4). Every third section of the C7–C8 spinal segment from each rat was used for GFAP immunohistochemistry (IHC), nNOS immunofluorescence (IF), or a neutral red stain (Sigma, St. Louis, MO, USA) following procedures from previous publications [9, 10]. Briefly, sections were washed three times in 0.01 M PBS for 10 min. For GFAP IHC, the sections were incubated in 0.3% peroxide in methanol (100%) at room temperature for 15 min to quench endogenous peroxidase activity. Before incubation with the primary antibodies, the sections were blocked with 3% BSA and 0.3% Triton X-100 in 0.01 M PBS at room temperature for 30 min. Then, the sections were incubated with the primary antibodies (Santa Cruz Biotechnology, Dallas, Texas, USA), including mouse anti-GFAP (1:2000;) and mouse anti-nNOS (1:500), in PBS for 72 h at 4°C. For nNOS IF, the sections were washed 3 times in PBS for 10 min per wash, and the sections were incubated at room temperature for 2 h in the dark with FITC conjugated anti-mouse IgG (1:2000, Sigma, St. Louis, MO, USA). After the sections were washed thoroughly, the sections were mounted on glass slides and coverslipped in antifade mounting media (50% glycerin in 0.5 M buffer bicarbonate, pH 9.5). For GFAP IHC, the sections were incubated in biotinylated anti-mouse IgG (1:500, Sigma, St. Louis, MO, USA) at room temperature for 2 h, then rinsed and incubated with ABC reagents (1:500, Boster, Wuhan, China) at room temperature for 45 min. The sections were washed thoroughly and incubated in 0.05% diaminobenzidine (DAB, Sigma, St. Louis, MO, USA) with 0.01‰ H_2_O_2_ for 3–5 min until a brown reaction product was observed. For the control slides, either the primary or secondary antibodies were omitted.

The quantitative studies of nNOS and GFAP immunoreactivity and the survival of spinal motoneurons were carried out with fluorescence microscopy (Carl Zeiss, Germany). The investigators were blinded to the animal subgroups to avoid bias. The data for each parameter in each subgroup were obtained from ten sections per animal for five rats. Spinal motoneurons showing cytoplasm stained with the nNOS antibody and a visible nucleus were counted under a 20× objective lens [23, 24]. The number of nNOS positive motoneurons in the ipsilateral ventral horn was expressed as a percentage of the surviving motoneurons in the ipsilateral ventral horn in the same section. The number of nNOS-positive motoneurons and the surviving motoneurons in each rat was expressed as the mean number of neurons counted in 10 serial sections. The quantitative assessment of GFAP immunoreactivity was carried out by determining the density of the GFAP reaction and using the pixel histogram generated for each original image by Image-Pro Plus 6.0 (Media Cybernetics, MD, USA). The product of the pixel number and pixel intensity value (0–255) was computed and summed for the entire image. The intensity of the GFAP immunoreactivity was expressed as the ratio of the ipsilateral ventral horn GFAP percentage to that of the contralateral ventral horn in 10 serial sections. The quantitative assessment of the survival ratio for the ventral horn motoneurons was conducted by counting surviving motoneurons in the neutral red stained slides. The spinal motoneurons showed visible nuclei on both the contralateral and the ipsilateral ventral horns, and the motoneurons were counted under a 10× objective lens as described in previous studies [23, 24]. The number of surviving motoneurons in the ipsilateral ventral horn was expressed as the percentage of surviving motoneurons in the contralateral ventral horn of the same section in 10 serial sections. Investigators were blinded to the animal group for motoneuron counting to avoid bias. The data for each parameter in each subgroup were obtained from ten sections per animal for five rats.

### Statistical Analyses

The data were expressed as the mean±SD. Statistical calculation and data handling were performed using SPSS software (version 16.0, SPSS Inc.) by an investigator who was blind to the animal groups. A difference between groups was considered statistically significant if the *P* value was less than 0.05. Comparisons across the 3 treatments over time for the I/C ratios of the spinal cord or the intervertebral foramens were performed using 2-way repeated measures analysis of variance (ANOVA), Post-hoc tests were performed when multivariate interaction effects were found. We were only interested in the differences among 3 treatments at each time-point. Multiple group comparisons were analysed with a one-way ANOVA followed by the Bonferroni post-hoc test. Multiple group comparisons for the GFAP intensity, nNOS-positive motoneurons, or surviving motoneurons in the ipsilateral ventral horns were analysed with a one-way ANOVA followed by the Bonferroni post-hoc test. The correlation of the I/C ratio of the ipsilateral sides of the spinal cord, the density of the GFAP immunoreaction in the ipsilateral ventral horns, the number of nNOS positive motoneurons in the ipsilateral ventral horn, and the survival ratio of the ipsilateral ventral horn motoneurons were assessed with the Spearman correlation coefficient.

## Results

### ^18^F-FDG Micro-PET/CT Images in the Cervical Spinal Cords of BPRA-Injured Rats

The results of the micro-PET/CT imaging showed an increased focal ^18^F-FDG uptake in the spinal cords of all BPRA rats from 3 d to 8 w post-injury, but there was no apparent change in ^18^F-FDG uptake in the control rats (Fig 1A–1C). In the avulsion-injured rats, a large amount of ^18^F-FDG accumulated in the cervical spinal segments was found in tomographic sections of the spinal cord, but similar accumulation was not found in the thoracic spinal segments (Fig 1). In Av+NS rats at 3 d post-injury, the average ROI in the thoracic spinal cord was 0.462±0.118, which was similar to that in the control rats, 0.443±0.060. However, BPRA led to a remarkable increase in ^18^F-FDG measures in the corresponding cervical spinal cord. The average uptake within the ROI was 0.666±0.144 in the ipsilateral side of the spinal cord and 0.639±0.134 in the contralateral side. When the cervical and the thoracic spinal cord were compared within the same rat in the Av+NS group at 3 d, the average uptake within the ROI for both the ipsilateral and the contralateral sides of the cervical spinal cord increased, but only the average uptake within the ROI of the ipsilateral cervical spinal cord was significantly higher than the average uptake within the ROI of the thoracic spinal cord (P<0.05). To eliminate the influence of ROI measurement of nearby structures, we used the ROIs of the contralateral side to normalize the ROIs in the ipsilateral side of the same spinal cord and recorded this measurement as the I/C ratio in the remaining experiments.

In the control rats (Fig 1B), the ^18^F-FDG PET images showed a similar pattern during different time points post-injury. However, the distribution of the ^18^F-FDG in C7–C8 cross sections was not equal among different tissues. According to the scale tomography for the signal intensity, the highest uptake of ^18^F-FDG occurred at the centre where the inner grey matter and outer white matter are localized. The spinal cord tissue showed a circular red-coloured area surrounding a yellow-coloured point, which was the central canal. The lowest uptake of ^18^F-FDG occurred at the bony structures, including the intervertebral foramen, which showed a white to blue colour in the outermost peripheral circle of the section. Between the red-coloured spinal cord tissue and the white-blue-coloured bony cervical vertebra, the thick, green-coloured outer layer close to the bony cervical vertebra and the thin yellow-coloured inner layer closely adhered to the spinal cord tissue.

### ^18^F-FDG Micro-PET/CT Images and Histological Responses at the Ipsilateral Intervertebral Foramen

In the present study, the microsurgery for inducing BPRA was carried out at the ipsilateral intervertebral foramen. The results of the ^18^F-FDG micro-PET/CT images of the rats’ C7–C8 spinal segments of both the Av+NS and the Av+GM1 groups from 3 d to 4 w post-injury showed that the most obvious increases in ^18^F-FDG uptake occurred at the ipsilateral intervertebral foramen compared to images from the control rats (Fig 1B). A group by time interaction effect was found for the I/C ratios of the intervetebral foramen (F = 9.996, p<0.001). A significant differences of the I/C ratios over time was found among the 3 different treatments (F = 76.649, p<0.001). After GM1 treatment, Av+GM1 rats had lower I/C ratio in the ipsilateral intervetebral foramen compared to the Av+NS rats (p < 0.001), but higher I/C ratio than the Sham controls (p = 0.001). Comparison among 3 treatments at the same time point showed significant differences at 3 d, 1 w, 2 w (all *P*<0.001), but not at 4 w and 8 w (all *P*>0.05) post-injury (Fig 1C). The results showed significantly higher I/C ratios in the Av+NS group compared to the ratios in the control group at 3 d, 1 w, and 2 w post-injury (all *P*<0.001, Fig 1C). At 4 w and 8 w post-injury, there were no significant differences between these two groups in the I/C ratios for the intervertebral foramen (all *P*>0.05, Fig 1C).

The histological and cytological responses of the C8 nerve roots in avulsed and un-avulsed rats were checked with a standard HE stain to confirm changes in ^18^F-FDG radioactivity at the intervertebral foramen (Fig 1E). In the control rats, the dorsal and ventral roots were well organized and completely separated from the nerve sheaths. In the dorsal root ganglia, the large neurons were grouped and separated by the nerve fasciculi with well-organized axons and sparse nuclear staining in glial cells. In the ventral root, there were approximately 3–4 rootlets enveloped by the perineurium, and well-organized axons occupied each rootlet. However, in all the avulsion-injured rats, there were no dorsal root ganglia because they had been removed during surgery, and almost all the ventral roots had disappeared. The histological structures of the distal end of the avulsed C8 spinal roots showed typical Wallerian degeneration. At 3 d post-injury, the most obvious change was the degeneration of the myelin surrounding the axons, including oedema, breakdown of myelin sheaths, phagocytosis within the myelin, and disorganized cellular patterns. Furthermore, increases in vessel calibre, vessel proliferation, local necrosis, bleeding, and glial reactions also occurred. At 1 w to 2 w post-injury, the size of the avulsed spinal roots was much smaller with very few degenerated axons and fibrosis.

### ^18^F-FDG Micro-PET/CT Images and Neuropathology of the Ipsilateral Spinal Cord Tissues

In the ^18^F-FDG micro-PET/CT images (Fig 1B), the spinal cord tissue appeared as a yellow-to-red-coloured mass at the centre of the C7–C8 cross sections of segments from the avulsion-injured rats. Higher ^18^F-FDG radioactivity also occurred between the peripheral bony structure and the central spinal cord tissue from 3 d to 8 w post-injury. Because unilateral BPRA-induced spinal motoneuron death occurred only in the ipsilateral cervical spinal cords, we further quantified the ROIs on the ipsilateral side of the C7–C8 spinal segments by using the contralateral side in the same cross section as a control (Fig 1D). A group by time interaction effect was found for the I/C ratios of the spinal cord (F = 3.866, p = 0.02). A significant differences of the I/C ratios over time was found among the 3 different treatments (F = 6.668, p = 0.03). After GM1 treatment, Av+GM1 rats had lower I/C ratio in the ipsilateral spinal cord compared to the Av+NS rats (p = 0.026). Comparison among 3 treatments at the same time point showed significant differences at 1 w (*P* = 0.037), 2 w (*P* = 0.003), 4 w (*P* = 0.015) post-injury (Fig 1D). The result showed that the I/C ratios were significantly higher in the Av+NS group at 1 w and 2 w (all P<0.05) but not significantly higher at 3 d and 4 w post-injury (all P>0.05) compared the control group (Fig 1D). There were no significant differences in the I/C ratios at 3 d and 8 w post-injury among the three experimental groups (all P>0.05, Fig 1D).

**Fig 2:**
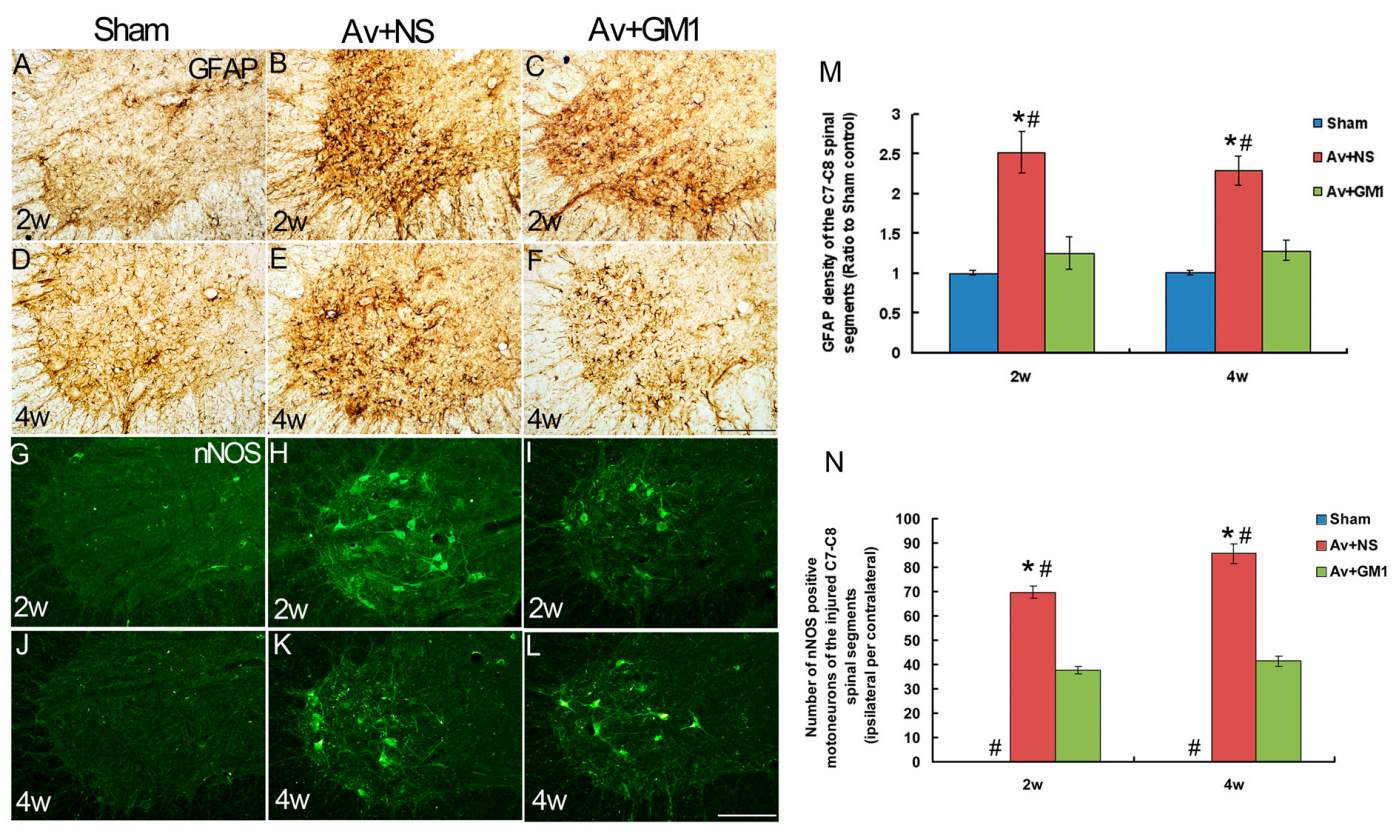
GM1 treatment reduces astrocyte reactions and inhibits nNOS expression in affected motoneurons (MNs) in the ipsilateral ventral horns of C7–C8 spinal cord at 2 w and 4 w post-injury. Scale bar = 200 μm. (A-F): Representative microphotographs showing avulsion-induced astrocyte reactions in the ventral horns with GFAP immunohistochemistry. (G-L): Representative microphotographs showing nNOS expression in ventral horn motoneurons with nNOS immunofluorescence. (M): Histograms showing average GFAP densities in the ventral horn as the ratio of the ipsilateral ventral horn percentage to the percentage of the contralateral ventral horn in each section of BPRA-injured or GM1-treated spinal cords. The density of the GFAP immunostaining significantly increased in the Av+NS group compared to the Av+GM1 group. (N) The number of nNOS-positive motoneurons of the injured spinal cord significantly increased in the Av+NS group compared to the Av+GM1 group. #*p* < 0.05 compared to the Av+GM1 group, * *p* < 0.05 compared to the Sham group.

In the present study, GFAP IHC (Fig 2A–2F) was used to assess BPRA-induced glial reactions at 2 w and 4 w post-injury. Compared to the general morphology of astrocytes in C7–C8 spinal sections in the control group (Fig 2A and 2D), the astrocytes had larger cell bodies and short processes in all of the avulsion-injured rats. The astrocytes were also distributed widely in the peripheral circle of the white matter on the ipsilateral side and accumulated in the grey matter of the ipsilateral ventral horns (Fig 2B and 2E). Quantitative analysis of the percentage of GFAP intensity in the ipsilateral horns compared to the contralateral ventral horns revealed significantly higher GFAP intensity in the Av+NS group at 2 w and 4 w post-injury compared the control group (Fig 2M).

In this study, nNOS IF (Fig 2G–2L) was used to detect spinal motoneurons affected by BPRA at 2 w and 4 w post-injury. The results showed there was no nNOS expression in the ventral horn motoneurons in the control rats at any time point (Fig 2G and 2J). In the Av+NS rats, avulsion induced remarkable expression of nNOS protein in ventral horn motoneurons at 2 w (Fig 2H) and 4 w (Fig 2K) post-injury. Quantitative analysis showed that the number of nNOS-positive motoneurons in the Av+NS group were 69.70%±2.46% at 2 w and 85.69%±3.96% at 4 w.

**Fig 3:**
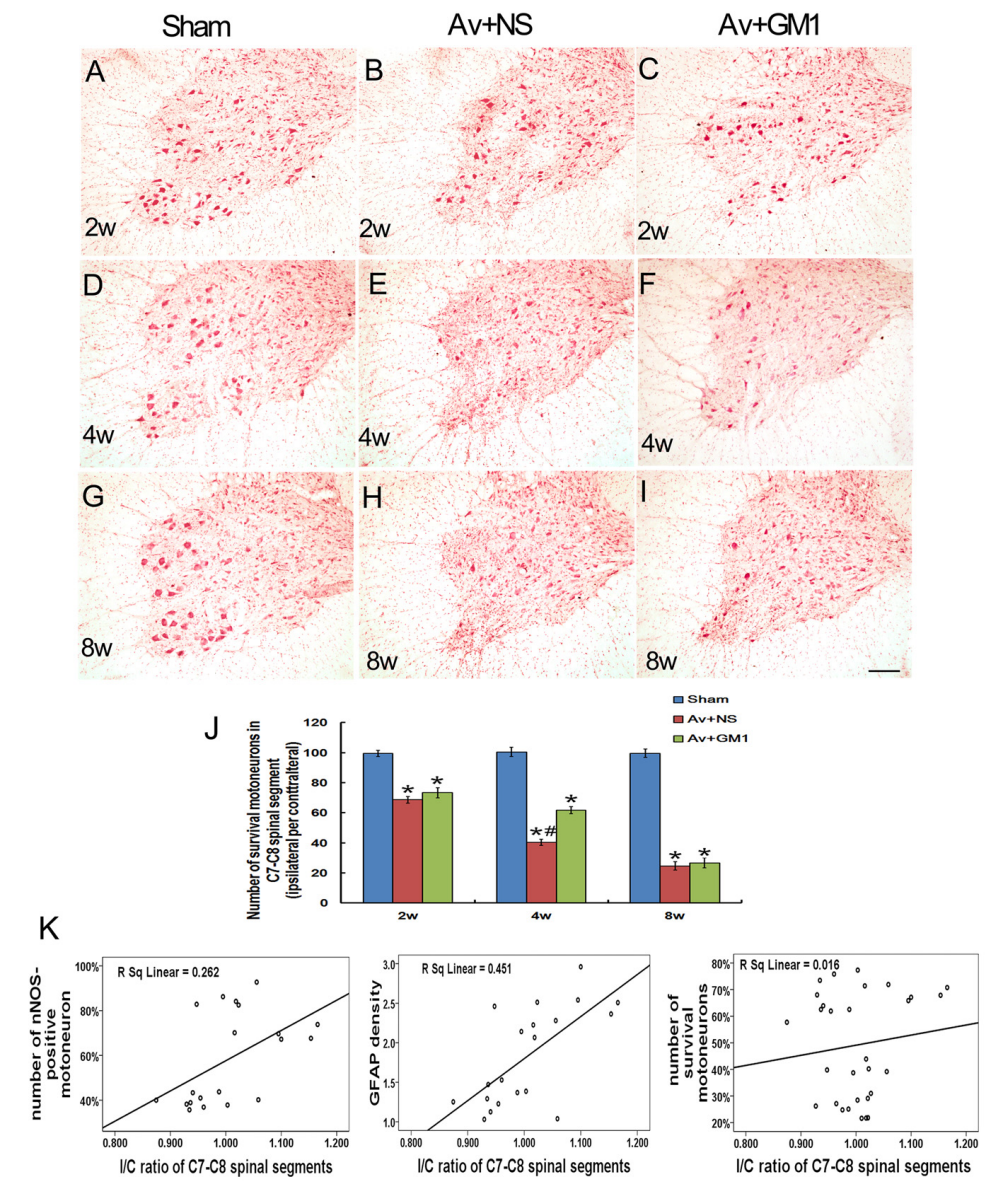
GM1 enhanced the survival of injured motoneurons and the correlation of ^18^F-FDG uptake with the GFAP density and number of nNOS-positive and surviving motoneurons in the ipsilateral C7–C8 spinal cord. (A–I) Representative microphotographs of the surviving motoneurons stained with neutral red in the C8 spinal ventral horns at 2 w, 4 w and 8 w post-injury. Scale bar = 200 μm. J: Quantitative study of the survival rate showed significantly lower survival for motoneurons in the Av+NS group and the Av+GM1 group. The survival rate was significantly higher in the GM1-treated rats compared to the avulsion group. *p < 0.05 compared to the Sham group, #*p* < 0.05 compared to the Av+GM1 group. K: Correlation analysis between the I/C ratio of the ipsilateral sides of the spinal cord with GFAP intensity, nNOS-positive motoneurons, or surviving motoneurons in the ipsilateral ventral horns at 2 w and 4 w post-injury. The I/C ratio in the ^**18**^F-FDG uptake was positively correlated with the density of the GFAP intensity (*R* = 0.672, *P*<0.001) and the number of nNOS positive motoneurons in the ipsilateral ventral horn (*R* = 0.525, *P*<0.05), but the ratio was not correlated with the survival ratio for ipsilateral ventral horn motoneurons (*R* = 0.086, *P*>0.05).

Survival of motoneurons in the ipsilateral C7–C8 sections was determined with images of the neutral red stained tissue (Fig 3) at 2 w (Fig 3A–3C), 4 w (Fig 3D–3F) and 8 w (Fig 3G–3I) post-injury. With the quantitative comparison between the control group and the Av+NS group, the survival rates were (99.55%±2.01% *vs* 68.62%±2.25% at 2 w; 100.50%±3.09% *vs* 40.37%±1.94% at 4 w; and 99.58%±2.82% *vs* 24.67%±2.74% at 8 w) significantly lower in the Av+NS group than in the control group at each time point (all *P*<0.05, Fig 3J).

### GM1 Treatment Reduced Avulsion-Induced Neuropathology and Decreased Uptake of ^18^F-FDG in Micro-PET/CT Images

In the Av+GM1 rats, both the site-specific and time-specific patterns of the ^18^F-FDG uptake in the cervical spinal cord were similar to the patterns in the Av+NS group. However, GM1 treatment yielded significantly lower I/C ratios at the intervertebral foramen at 1 w and 2 w compared to the Av+NS group at the same time point (all P<0.001). The I/C ratios were still significantly higher at 3 d and 1 w compared to the control group (all P<0.001, Fig 1C). Histological examination of the avulsed spinal roots at the site of the intervertebral foramen showed less bleeding and earlier astrocyte gliosis in the Av+GM1 group compared to the Av+NS group (Fig 1E). In the spinal cord tissue, significantly lower I/C ratios were measured at 2 w and 4 w in the Av+GM1 group compared to the Av+NS group at the same time point (all P<0.05, Fig 1D). Furthermore, the I/C ratio was significantly lower in the Av+GM1 group compared to the control group at 4 w post-injury (P<0.05, Fig 1D).

The remarkable inhibition of BPRA-induced glial reactions of the GM1 treatment was demonstrated with GFAP IHC (Fig 2C and 2F). Quantitative analysis showed the GFAP intensity significantly decreased compared to the intensity of the Av+NS group (all *P*<0.05) at 2 w and 4 w post-injury (Fig 2M). Furthermore, there were no significant differences in the GFAP intensity between the Av+GM1 group and the control group (all *P>*0.05). Moreover, GM1 drug treatment also inhibited avulsion-induced nNOS expression in spinal motoneurons. Quantitative analysis showed that the number of nNOS-positive motoneurons in the Av+GM1 group was 37.76%±1.57% at 2 w and 41.38±1.98% at 4 w, significant decreases compared to the Av+NS group at the same time points (all *P*<0.05, Fig 2N). With GM1 treatment, more affected motoneurons survived the BPRA injury (Fig 3C, 3F and 3I) with survival rates of 73.33%±3.42% at 2 w, 61.74%±2.22% at 4 w, and 26.74%±3.46% at 8 w post-injury (Fig 3J). In the Av+GM1 group, the survival rates of the motoneurons were significantly higher than those in the Av+NS subgroup at 4 w post-injury (*P*<0.05, Fig 3J).

To detect the reasons for the significant increase in ^18^F-FDG radioactivity in PET/CT images of the Av+NS and Av+GM1 subgroups, a correlation analysis was carried out between the I/C ratio of the ipsilateral sides of the spinal cord for GFAP intensity, nNOS-positive motoneurons, or surviving motoneurons in the ipsilateral ventral horns at 2 w and 4 w post-injury (Fig 3K). The correlation analysis showed that the I/C ratio in the ^18^F-FDG PET/CT images of the ipsilateral sides of the spinal cord was positively correlated with both the density of GFAP intensity in the ipsilateral ventral horns (*R* = 0.672, *P*<0.001) and the number of nNOS positive motoneurons in the ipsilateral ventral horn (*R* = 0.525, *P*<0.05), but the ratio was not correlated with the survival ratio of the ipsilateral ventral horn motoneurons (*R* = 0.086, *P*>0.05).

## Discussion

The data in the present study showed that unilateral brachial root avulsion induces a site-specific and time-specific pattern of ^18^F-FDG uptake in the cervical spinal cord. Within the first two weeks post-injury, significantly higher ^18^F-FDG radioactivity was detected in the ipsilateral intervertebral foramen (the site of the root avulsion). Furthermore, the avulsion-injured ipsilateral cervical spinal cord also showed high ^18^F-FDG radioactivity. The results suggest that altered FDG metabolism in the avulsed nerve roots at the ipsilateral intervertebral foramens and the corresponding ipsilateral spinal cord changes over time. Neuropathological studies demonstrated the Wallerian degeneration of the distal ends of the avulsed spinal nerve roots at the intervertebral foramen. The high uptake of ^18^F-FDG in the spinal cord tissue was significantly correlated with BPRA-induced astrocyte reactions in both grey and white matter as well as with the de novo nNOS protein expression in the ipsilateral ventral horn motoneurons.

In brachial plexus root avulsion, the primary injury involves severance of the ventral and dorsal roots from the surface of the corresponding spinal cord, the rupture of the dura mater, and the rupture of the radical vessels accompanying the nerve roots [25, 26]. In the present study, all these primary injuries occur during BPRA surgery. More importantly, we found high ^18^F-FDG uptake in the ipsilateral intervertebral foramen within the first 2 weeks with micro-PET/CT. Previous studies have demonstrated that the local bleeding in the dura sac, bleeding in the subarachnoid spaces and breakdown of the brain-blood barrier through rupture of the radical vessels are followed by vessel enlargement and proliferation of the spinal cord injury from the ventral root avulsion [27–29]. High glucose metabolism during this type of vascular response in cerebral vessel diseases and trauma of the nervous system have been found to contribute to higher uptake of ^18^F-FDG [18, 30–32]. This conclusion is supported by the data from this study, which showed persistently high uptake of the ^18^F-FDG in the ipsilateral intervertebral foramen surrounding the corresponding spinal cord of the avulsion-injured rats. However, the high ^18^F-FDG uptake at the ipsilateral intervertebral foramen reflected not only primary injuries from BPRA surgery but also the histological responses of the distal ends of avulsed nerve roots within the first 2 weeks. The histological changes of the avulsed nerve roots have shown the increase in the vessel calibre, vessel proliferation, local necrosis, and bleeding. The nerve fibres showed typical Wallerian degeneration at early stages, including oedema, breakdown of myelin sheaths, phagocytosis, and glial reactions. Previous studies have demonstrated higher FDG uptake in vessel walls due to macrophage activation and cellular proliferation [32, 33]. The overexpression of a GLUT1 receptor subtype due to macrophage, lymphocyte and neutrophil activation in these pathological vessels may contribute to FDG uptake [33]. Moreover, the gliosis in the avulsed roots also contributes to higher FDG uptake. This result was supported by previous studies, which demonstrated increased uptake of FDG through PET in the neurofibroma of peripheral nerves [34, 35].

In the present study, GFAP expression increased in white matter and grey matter of the spinal cord, and the increase was most obvious around the injured motoneurons. The progressive increase in astrocyte staining in the ventral horn after brachial root avulsion coincides with previous experiments [9, 36]. Previous studies have demonstrated that root avulsion injury can trigger activation of astrocytes, microglia, and macrophages surrounding the affected ventral horn motoneurons [5, 8, 9, 36]. Yuan et al. found brain macrophages and reactive microglia in the lesioned area, as well as in the area surrounding the motoneurons, through a combination of ED1 and OX-42 IHC, but the microglial reaction was sustained only for the first 7 days following BPRA [9]. Many previous studies have also demonstrated activation of the microglia in response to central nervous system injuries at a very early stage, which is a response that often precedes reactions of any other cell type in the brain [37, 38]. In contrast, other studies also found injury-induced activation of microglia at later stages when the affected central neurons had died [39, 40]. In the present study, the significant BPRA-induced increase in ^18^F-FDG uptake in the spinal cord tissue occurred at 2 w and 4 w, which is the middle stage of BPRA. Therefore, we did not investigate microglia or macrophages but focused on detecting astrocyte activation with GFAP IHC. As previously reported, GFAP is an intermediate filament protein in the soma and dendrites of astrocytes. GFAP has house-keeping functions in astrocytes, including maintaining the microenvironment for neurons and glucose metabolism of the central nervous system, which is primarily controlled by astrocytes [41, 42]. Astrocytes phosphorylate 75% of the glucose that enters the parenchyma through a glucose transporter (GLUT1) [43–47]. The present data support these previous results by showing a positive correlation between the I/C ratio in ^18^F-FDG PET/CT imaging of the ipsilateral sides of the spinal cord and GFAP intensity in the ipsilateral ventral horns.

The typical neuropathological changes from BPRA, such as de novo nNOS expression inside the affected motoneurons [5, 10–13, 23, 24], were found in the present study. Avulsion-induced de novo expression of nNOS inside motoneurons may facilitate the survival of injured motoneurons [23, 48] at the early stages of the injury. Based on the small PET/CT results, we believe that the increased biochemical progress of nNOS protein synthesis in affected motoneurons may contribute to higher ^18^F-FDG uptake in the ipsilateral side of the spinal cord. Moreover, the overexpression of nNOS results in secondary oxidative stress of the injured spinal cord and correlates with motoneuron apoptosis [11,12]. The pathological changes from the oxidative stress and apoptosis have been considered as the energy-consuming processes [49] that could be accompanied by an increase in ^18^F-FDG [50]. Our data showed that the I/C ratio in the ^18^F-FDG PET/CT imaging of the ipsilateral sides of the spinal cord was significantly and positively correlated with the number of nNOS positive motoneurons in the ipsilateral ventral horn, which suggests that avulsion-induced oxidative stress can be accompanied by ^18^F-FDG uptake.

The time-course and severity of motoneuron loss in the present study coincided with those of a previous study [10]. The previous study showed atrophy and necrosis of anterior grey horn cells in chronic compression of the spinal cord, which led to the loss of glucose-consuming neurons and a decrease in FDG uptake in the spinal cord [51, 52]. Neuron loss was thought to decrease brain tissue glucose uptake in certain regions of the ipsilateral hemisphere suffering from brain trauma, such as during cerebral ischemic stroke [29]. However, in the present study, neuronal loss accompanied higher FDG uptake within the first two weeks. The molecular and cellular responses in the ipsilateral side of the cervical spinal cord contributed to higher FDG in BPRA. Our data showed that the de novo expression of nNOS protein and the astrocyte reactions were positively correlated with higher FDG uptake within the first two weeks. The loss of motoneurons might not be sufficient to cause lower FDG uptake in the ipsilateral spinal cord. Previous studies considered intraspinal FDG uptake, which was physiologically confirmed to be in the artery of the spinal cord [53]. Our present data support these previous studies, which showed higher FDG uptake in the cervical spinal cord compared to the thoracic segments. Anatomically, the arterial supplies of the cervical spinal cord come from the anterior and posterior spinal arteries as well as from the radical artery, which is larger than the arterial supply from the anterior spinal artery and the radical artery supporting the thoracic spinal cord. Compared to the thoracic spinal cord, higher FDG uptake in the cervical spinal cord has also been confirmed in a clinical scenario [54]. During BPRA surgery, rupture of the radical artery caused partial bleeding, as shown in the histology examination from the present study.

In the present study, we used micro-PET/CT to diagnose spinal cord injuries by assessing extremely small regions of the ipsilateral spinal cord. To decrease the influence of FDG uptake from nearby structures on FDG measures in spinal cord tissues, we used the contralateral side to normalize FDG measures in the ipsilateral side. Our data suggest that ^18^F-FDG micro-PET/CT is a good method for evaluating injuries in spinal nerve roots and the spinal cord. In diagnosing the local structures of small animals in vivo, the tracers for PET and other imaging modalities, such as magnetic resonance imaging (MRI) and ultrasound, were studied previously. More recent ultrasound devices with very high frequencies (40–55 MHz) have been specifically designed to image small animals with spatial resolutions as low as 30 microns per pixel [55]. MRI is considered to be the most accurate imaging technique because of the excellent soft-tissue contrast resolution [56]. In comparison to ultrasound and MRI, FDG PET is a functional method based on glucose metabolism. FDG-PET/CT is considered to be the superior method for detecting active functional responses to trauma, infection and degenerative inflammation, but this approach can only diagnosis relatively limited areas of abnormal metabolic activity [30]. Most recently, imaging biomarkers for PET have been shown to be potentially capable of assessing the extent of central nervous system diseases, such as ^18^F-MNI-659 for assessing early motor signs of Huntington disease [57], ^18^F-DPA-714 for assessing neuroinflammation in the human brain [58] and ^18^F-GE-180 for revealing the presence of activated microglia in both grey and white matter [59]. Our present data indicate that ^18^F-FDG can be used to assess the severity of brachial plexus root avulsion at the intervertebral foramen, and ^18^F-FDG can also be used to assess avulsion-induced secondary injury in the spinal cord.

Another important finding in the present study is that GM1 therapy can reduce glial cell reactions, inhibit nNOS overexpression, and rescue some of the motoneurons in the local spinal cord. GM1 treatment immediately following brachial root avulsion can reduce the primary injuries in the avulsed roots. The protective effect of GM1 on the avulsion-injured motoneurons coincided with the results of previous studies on GM1 therapy in the central nervous system [60–65]. Moreover, the effect of GM1 on the protection of injured central neurons may be related to the inhibition of oxidative stress [60, 61] and glial reactions as well as to neuroinflammation in the local brain structure [62]. This theory is further supported by the present data. Moreover, previous studies also demonstrated that GM1 contributes to neuronal survival through the following mechanisms: (1) exogenous GM1 can cross the blood-brain barrier to protect cell membranes through rescue of Na^+^–K^+^ ATPase and Ca^2+^–Mg^2+^ ATPase activity to maintain a balance of ions and reduce water accumulation within the neurons [60], which may contribute to the lower FDG uptake in the Av+GM1 rats compared to the Av+NS rats in the present study; (2) GM1 promotes the expression of Bcl-2 and inhibits the expression of Bax [63]; and (3) GM1 benefits neuronal recovery by promoting the effects of the neurotropic factor, reducing secondary neurotoxicity, regulating synaptic signalling, improving nerve conduction velocity, and repairing damage in the neurons [64, 65].

## Conclusion

Serial ^18^F-FDG small-animal PET/CT imaging demonstrated higher metabolism in lesion sites and showed that there was ipsilateral spinal cord involvement in the earlier stages of brachial plexus root avulsion. Avulsion-induced astrocyte activation in injured spinal cord and overexpression of the nNOS protein in the affected motoneurons contributed to higher radioactivity in vivo in the ^18^F-FDG small-animal PET/CT images. We concluded that ^18^F-FDG PET/CT imaging can be a useful tool for the assessment of primary and secondary spinal cord injuries as well as spinal root avulsion injuries. The optimal assessment of a spinal cord injury is beneficial for timely surgical repair of brachial plexus root avulsion in clinical settings. Furthermore, our present data indicated that GM1 is an effective drug for reducing primary and secondary spinal cord injuries following root avulsion.
